# Automated Long-Term EEG Review: Fast and Precise Analysis in Critical Care Patients

**DOI:** 10.3389/fneur.2018.00454

**Published:** 2018-06-19

**Authors:** Johannes P. Koren, Johannes Herta, Franz Fürbass, Susanne Pirker, Veronika Reiner-Deitemyer, Franz Riederer, Julia Flechsenhar, Manfred Hartmann, Tilmann Kluge, Christoph Baumgartner

**Affiliations:** ^1^Karl Landsteiner Institute for Clinical Epilepsy Research and Cognitive Neurology, Vienna, Austria; ^2^Department of Neurology, General Hospital Hietzing With Neurological Center Rosenhügel, Vienna, Austria; ^3^Department of Neurosurgery, Medical University of Vienna, Vienna, Austria; ^4^Center for Health and Bioresources, AIT Austrian Institute of Technology GmbH, Vienna, Austria; ^5^Epilepsie-Zentrum Berlin-Brandenburg, Ev. Krankenhaus Königin Elisabeth Herzberge, Berlin, Germany; ^6^Medical Faculty, Sigmund Freud University, Vienna, Austria

**Keywords:** neurotrend, intensive care unit, continuous EEG, non-convulsive seizures, status epilepticus, standardized critical care EEG terminology

## Abstract

**Background:** Ongoing or recurrent seizure activity without prominent motor features is a common burden in neurological critical care patients and people with epilepsy during ICU stays. Continuous EEG (CEEG) is the gold standard for detecting ongoing ictal EEG patterns and monitoring functional brain activity. However CEEG review is very demanding and time consuming. The purpose of the present multirater, EEG expert reviewer study, is to test and assess the clinical feasibility of an automatic EEG pattern detection method (Neurotrend).

**Methods:** Four board certified EEG reviewers used Neurotrend to annotate 76 CEEG datasets à 6 h (in total 456 h of EEG) for rhythmic and periodic EEG patterns (RPP), unequivocal ictal EEG patterns and burst suppression. All reviewers had a predefined time limit of 5 min (± 2 min) per CEEG dataset and were compared to a predefined gold standard (conventional EEG review with unlimited time). Subanalysis of specific features of RPP was conducted as well. We used Gwet's AC_1_ and AC_2_ coefficients to calculate interrater agreement (IRA) and multirater agreement (MRA). Also, we determined individual performance measures for unequivocal ictal EEG patterns and burst suppression. Bonferroni-Holmes correction for multiple testing was applied to all statistical tests.

**Results:** Mean review time was 3.3 min (± 1.9 min) per CEEG dataset. We found substantial IRA for unequivocal ictal EEG patterns (0.61–0.79; mean sensitivity 86.8%; mean specificity 82.2%, *p* < 0.001) and burst suppression (0.68–0.71; mean sensitivity 96.7%; mean specificity 76.9% *p* < 0.001). Two reviewers showed substantial IRA for RPP (0.68–0.72), whereas the other two showed moderate agreement (0.45–0.54), compared to the gold standard (*p* < 0.001). MRA showed almost perfect agreement for burst suppression (0.86) and moderate agreement for RPP (0.54) and unequivocal ictal EEG patterns (0.57).

**Conclusions:** We demonstrated the clinical feasibility of an automatic critical care EEG pattern detection method on two levels: (1) reasonable high agreement compared to the gold standard, (2) reasonable short review times compared to previously reported EEG review times with conventional EEG analysis.

## Introduction

Nonconvulsive seizures (NCS) and nonconvulsive status epilepticus (NCSE) are a common burden for neurological critical care patients. People with epilepsy or epileptic encephalopathy often develop ongoing NCSE after status epilepticus (SE) with prominent motor activity (convulsive SE) (1–4). Functional outcome and prognosis may be worse in patients with ongoing NCSE due to increased metabolic demand and thus causing secondary brain damage (5–8). Recent studies show mortality of up to 40% in super-refractory SE and increased costs and length of stay associated with refractory course (9). Continuous EEG (CEEG) in neurological intensive care units is currently considered the gold standard for detecting NCS and NCSE as well as monitoring sedoanalgesia and induced burst suppression patterns in patients with refractory or super-refractory SE (10). However, CEEG is very labor-intensive and time consuming in terms of visual real time analysis in daily practice (11). Automatic analysis tools are a promising approach to solve this shortcoming of CEEG. Previous publications focused mostly on quantitative EEG analysis and showed seizure identification sensitivities of 43–94% (11–17).

Our study group developed an automated analysis software called NeuroTrend (NT) and previously described the mathematical and technical details of the software (18). In short, NT consists of several mathematical algorithms which detect rhythmic and periodic EEG patterns (RPP, i.e., periodic discharges, rhythmic delta activity and spike-and-wave complexes) according to the ACNS standardized critical care EEG terminology (SCCET) as well as faster rhythmic activity in the theta and alpha range. The core idea of NT is to give a smooth overview of up to 100 h of CEEG in a graphical user interface (GUI), visualizing automatic analysis results in a horizontal fashion. Raw EEG data of each detection result can be easily assessed and reviewed on a separate computer monitor. In this way EEG reviewers can focus on pre-analyzed episodes of interest.

NT showed high sensitivity for the detection of RPP in a previous study (19). Results of this study were critically reviewed and the software was further improved in terms of specificity. In a second study, NT was evaluated as bedside monitoring in intensive care nurses (non-EEG-expert reviewers). Herta et al. showed that multirater agreement (MRA) and interrater agreement (IRA) were almost perfect for spike-and-wave complexes, rhythmic delta activity, and burst suppression. Electrographic seizure patterns and periodic discharges showed substantial agreement (20).

The current study focuses on the clinical feasibility of NT as CEEG review tool. Specifically we hypothesized, that NT is a time saving method which detect relevant findings in CEEG with high accuracy. Therefore we conducted a multirater study with four board certified EEG reviewers (expert EEG reviewers) annotating CEEG datasets using NT with predefined time limits (5 min ± 2 min) and compared theses annotations with a predefined gold standard.

## Methods

**Figure 1:**
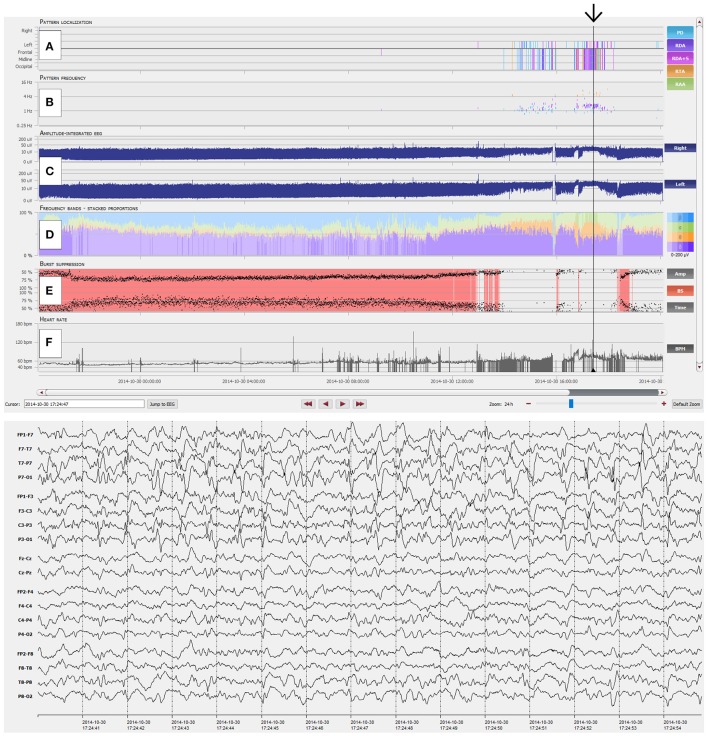
Overview of the NeuroTrend graphical user interface (GUI). **(A)** Automatic, color coded pattern detection (light blue: PD, periodic discharges; violet: RDA, rhythmic delta activity; pink: RDA+S, rhythmic delta activity plus superimposed spikes; orange: RTA, rhythmic theta activity; light green: RAA, rhythmic alpha activity); **(B)** Related frequencies of detected EEG patterns (the same color code as in A is used); **(C)** Amplitude integrated EEG for left and right hemisphere; **(D)** Frequency bands (beta-alpha-theta-delta) in a color coded (blue: beta; green: alpha; orange: theta; violet: delta), stacked proportion view (stronger colors signal higher amplitudes); **(E)** Burst suppression detection (continuous red markers signal presence of burst suppression); **(F)** Heart rate frequency plot. The black arrow highlights an EEG example of 1.5-2 c/s left hemispheric periodic discharges with superimposed rhythmic activity, which can be easily detected with the Neurotrend GUI.

**Figure 2:**
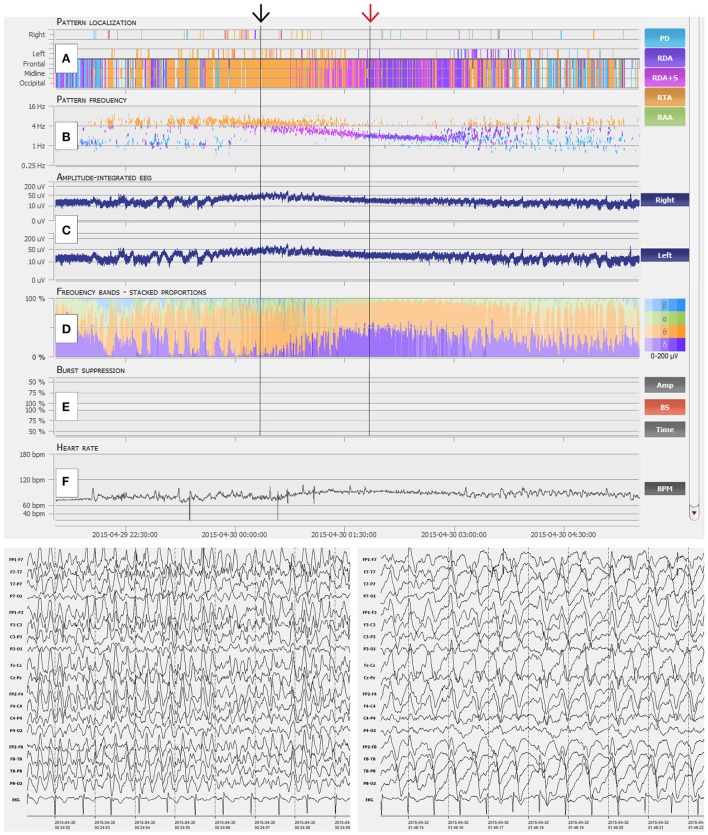
Interpretation of NeuroTrend. **(A)** Recurrent seizures are detected as generalized rhythmic theta activity (RTA, orange plots) between 22:30 and 00:00. Then ongoing seizure activity is displayed by ongoing detection of RTA until 01:30. Around 01:00 detection of generalized rhythmic delta activity (RDA, pink and violet plots) overlap with RTA and further increases until 03:00. **(B)** Related pattern frequency detection reveal clear cut seizures above 3 c/s between 22:30 and 01:30 (black arrow). Overlapping RDA show a steady decrease from 3.5 to 2 c/s (red arrow). **(C)** Amplitude integrated EEG shows increment and decrement over both hemispheres at the beginning of each seizure from 22:30 to 23:30. Then a steady increase over both hemispheres can be seen during ongoing seizure activity from 00:00 until 01:00. **(D)** Frequency bands show a dominance of theta activity during seizure activity and the overlap of theta and delta activity around 01:30. **(E)** No burst suppression was detected. **(F)** Heart rate does not really show a concordance to seizure activity. In synopsis, this example represent typical spatiotemporal evolution of electrographic seizure activity, which can be easily detected with the graphical user interface of Neurotrend.

We recruited four experienced, board certified EEG reviewers (SP, VR, FR, and JF) from our department to review 80 continuous EEG (CEEG) datasets of 20 critical care patients, each lasting 6 h, with an automatic EEG analysis software (Encevis, NeuroTrend, AIT Austrian Institute of Technology GmbH, Vienna, Austria; http://www.encevis.com). The NT setup for the current study consisted of an EEG viewer (computer monitor #1, 1920 × 1080 pixels) and the separate trending tool GUI (computer monitor #2, 1280 × 1024 pixels). Figures 1, 2 give an overview of the NT GUI. All reviewers had more than 5 years of EEG reading experience and were blinded to patient selection, quantity of negative controls and conclusions of other reviewers.

### Setup and training

All four reviewers had moderate experience with critical care EEG recordings (i.e., all four reviewers read critical care EEGs on a weekly basis) and none with the automatic EEG analysis software (Encevis, NeuroTrend). We therefore trained all reviewers prior to our study with a modified version of the Critical Care EEG Monitoring Research Consortium's Training Module, (ACNS SCEET Training Module, http://www.acns.org/practice/guidelines) (21), refreshed the knowledge about state-of-the-art nonconvulsive seizure (NCS) criteria (Salzburg Consensus Criteria) (22) and gave an introduction to NT and its GUI. The initial training phase lasted 1 h. Subsequently, 10 training datasets of continuous critical care EEGs (CCEEG) were provided to all four reviewers. This second phase of training lasted also 1 h. Training slides were provided for self-study but could not be used during review.

### EEG data

Twenty CCEEG datasets out of 98 consecutive monitored, neurological critical care patients were randomly selected using Microsoft Excel's random number generation function. No patient could be drawn twice. We tried to provide a reflection of the actual incidences of rhythmic and periodic EEG patterns seen in critical care EEG recordings in our monitored patients. Therefore, the selection process was as follows:

1. Six patients without any rhythmic or periodic EEG pattern were selected as negative controls
2. All patients with RPP and/or electrographic seizures were separated in to four pools according to their dominant EEG pattern (i.e., PD, RDA, SW, electrographic seizures). Because 14 patients had to be selected, we calculated the relative proportion within the RPP/electrographic seizure group for each pattern. We calculated a relative incidence of 51% for PD and therefore selected 7 patients with PD for the present study. Accordingly, we selected 4 patients with RDA (relative incidence of 27%), 2 patients with electrographic seizures (relative incidence of 13%) and 1 patient with SW (relative incidence of 9%).

All CEEGs were recorded with a Micromed EEG recording system (SystemPLUS Evolution 1.04.95, Micromed S.p.A., Veneto, Italy) using 21 electrodes placed according to the International 10-20 system with a sampling rate of 256 Hz. Patients with less than 19 surface electrodes due to operational wounds, less than 24 h CEEG duration, technical insufficient EEG data and training datasets were excluded from the selection process.

The first 24 h of each CEEG dataset of every patient was cut into four equal parts, each part lasting 6 h. Thus, 80 CEEG datasets à 6 h were obtained. These datasets were randomized and then used for the review process.

### Clinical data

All reviewers obtained a short written overview of the original medical history for each patient included in the study. Medication, original EEG reports, medical procedures after CEEG and clinical diagnosis were withheld.

### Review process

All four reviewers analyzed 80 randomized CEEG datasets with NT. In order to answer our hypothesis, we set a 5 min time limit for each dataset (i.e., 6 h of CEEG). This time limit could be extended to a maximum of 7 min. The exact review duration for each dataset was recorded.

Reviewers had to use predefined annotation sheets (Supplementary Material 1) and annotate each CEEG dataset separately. We used following items according to the ACNS SCEET (21): (1) Presence of rhythmic or periodic EEG patterns (yes/no) (2) if yes, what does the annotated pattern represent (Status epilepticus/electrographic seizure/no ictal activity) (3) Localization (Main Term 1; generalized/lateralized/bilateral independent) (4) Morphology (Main Term 2; electrographic seizure pattern/spike-and-wave complexes/rhythmic delta activity/periodic discharges) (5) Prevalence (>90%/50–89%/10–49%/1–9%) (6) Frequency (>3Hz/1-3Hz/<1Hz), (7) Trend (evolution/fluctuation/stationary) (8) Presence of burst-suppression (yes/no) and (9) EEG background activity (slowing, yes/no; localization, focal/generalized; duration, intermittent/continuous).

### Gold standard

Two independent clinical neurophysiologists (JK and JH) with substantial CCEEG reading experience reviewed all CEEG datasets prior to this study. Our general CCEEG review strategy was described elsewhere (23). In short all CEEGs were classified according to the ACNS SCCET (21) and NCS criteria proposed by Leitinger et al. (Salzburg Consensus Criteria) (22). If discrepancies in the classification of certain EEG patterns occurred between the two reviewers, a third board-certified electroencephalographer (CB) with substantial CCEEG reading experience was involved. The third reviewer was involved in approximately 30% of all CEEG datasets, mainly to clarify the morphology (Main Term 2) of rhythmic and periodic EEG patterns. Using this method, we obtained consensus agreements for all CEEG datasets. We considered this visual EEG review consensus agreement as gold standard for the present study.

### Statistical analysis

Differences of review times between reviewers were calculated per patient and per EEG dataset with the Kruskal-Wallis test, because the recorded review times did not show a normal distribution. Chi-square test was used for categorical and ordinal data.

For IRA we used Gwet's multirater agreement coefficients AC_1_ (for categorical data) and AC_2_ (for ordinal data) (24). Gwet's AC_1_ and AC_2_ solve some shortcomings of established kappa coefficients, i.e., reliable performance if several raters show high or low agreement or if the true prevalence of classes being rated is nonuniform (25–27). We calculated IRA of each reviewer and our defined gold standard for the following annotation items:

1. Presence of RPP defined as follows:
  - No pathologic EEG patterns according to ACNS SCCET Main Term #2 and NCS criteria (equals “rhythmic and periodic EEG patterns not present” in the annotation sheet)
  - Interictal EEG patterns according to ACNS SCEET Main Term #2 but not fulfilling NCS criteria (equals “rhythmic and periodic EEG patterns present” and one of the following items “spike-and-wave complexes (SW),” “rhythmic delta activity (RDA)” or “periodic discharges (PD)” and “no ictal activity” in the annotation sheet)
  - Ictal EEG patterns fulfilling NCS criteria (equals “rhythmic and periodic EEG patterns present” and “Status epilepticus” or “electrographic seizure” in the annotation sheet)
2. Presence of unequivocal ictal EEG patterns (yes/no) defined as ictal EEG patterns fulfilling NCS criteria (equals “rhythmic and periodic EEG patterns present” and “Status epilepticus” or “electrographic seizure” in the annotation sheet)
3. Presence of burst-suppression (yes/no) according to ACNS SCEET Background EEG defined as “burst-suppression present” in the annotation sheet.

We calculated unweighted MRA between all four reviewers for following annotations items:

1. Presence of RPP as defined in the IRA section
2. Presence of unequivocal ictal EEG patterns as defined in the IRA section
3. Presence of burst-suppression as defined in the IRA section

We performed a subanalysis of RPP according to ACNS SCCET Main Terms and Modifiers. Annotations without RPP were excluded in the following manner: if two or less out of four reviewers did not annotate RPP in a specific EEG dataset, then this dataset was excluded from further analysis. We used custom weighted analysis (further details are provided in the Supplementary Material 2) and calculated MRA of the remaining EEG datasets for the following items:

- Localization (Main Term #1) defined as localization of RPP (equals “rhythmic and periodic EEG patterns present” and one of the following items “generalized”, “lateralized” or “bilateral independent” in the annotation sheet).
- Morphology (Main Term #2) defined as morphology of RPP (equals “rhythmic and periodic EEG patterns present” and one of the following items “SW,” “RDA,” or “PD” in the annotation sheet)
- Prevalence (Modifier #1) defined as prevalence of RPP (equals “rhythmic and periodic EEG patterns present” and one of the following items “>90%,” “50–89%,” “10–49%,” or “1–9%” in the annotation sheet)
- Frequency (Modifier #3) defined as frequency of RPP (equals “rhythmic and periodic EEG patterns present” and one of the following items “>3 Hz,” “1–3 Hz” or “<1 Hz” in the annotation sheet)
- Trend (Modifier #9) defined as trend of RPP (equals “rhythmic and periodic EEG patterns present” and one of the following items “evolution,” “fluctuation,” or “stationary” in the annotation sheet)

Following categories were used to quantify IRA and MRA: slight agreement 0.01–0.20; fair agreement 0.20–0.40; moderate agreement 0.40–0.60; substantial agreement 0.60–0.80; and almost perfect agreement 0.80–1 (25, 28). Confidence intervals of 95% were calculated as well.

Performance analysis of individual reviewers compared to the gold standard was conducted as follows for unequivocal ictal EEG patterns and burst suppression: CEEG datasets with positive reviewer annotation for ictal EEG patterns/burst suppression and positive gold standard annotation for ictal EEG patterns/burst suppression were counted as true positive (TP). If the gold standard showed no annotation in CEEG datasets with reviewer annotations for ictal EEG patterns/burst suppression, than they were counted as false positive (FP). CEEG datasets without reviewer annotation for ictal EEG patterns/burst suppression and without gold standard annotation for ictal EEG patterns/burst suppression were counted as true negative (TN). If the gold standard showed an annotation for ictal EEG patterns/burst suppression in CEEG datasets without a reviewer annotation, than they were counted as false negative (FN). We then calculated sensitivity (TP/[TP+FN]) and specificity (TN/[TN+FP]).

Statistical analysis was performed using the commercially available statistical software SPSS (IBM SPSS Statistics Version 21), Microsoft Office Excel 2010 and 2013, quantpsy.org (interactive online statistical calculation tool) and AgreeStat 2015.6 (http://agreestat.com). Bonferroni-Holmes correction for multiple testing was applied to all statistical tests. Significance levels for all statistical tests were set at *p* < 0.05 after Bonferroni-Holmes correction.

## Results

Four CEEG datasets were excluded from the study because of technical issues and low data quality. Therefore, the remaining 76 datasets, 6 h of CEEG each, were annotated by all four reviewers (in total 456 h of EEG). Mean review time was 12 min (± 5.3 min) per patient and 3.3 min (± 1.9 min) per CEEG dataset. There was a statistical significant difference of individual review times per patient and per CEEG dataset between reviewers (Table 1).

**Table 1:** Mean review times of four independent EEG reviewers, who analyzed 76 continuous EEG segments of 20 critical care patients à 6 h.

	**REV-1**	**REV-2**	**REV-3**	**REV-4**	*P*-Value^*^
Review time in minutes per patient (mean ± standard deviation)	9.1 (± 6.0)	10.2 (± 5.1)	15.2 (± 4.6)	13.6 (± 5.6)	0.007
Review time in minutes per 6 h of continuous EEG (mean ± standard deviation)	2.5 (± 1.8)	2.8 (± 2.1)	4.0 (± 2.3)	3.8 (± 1.4)	<0.001

*All four EEG reviewers used an automatic detection software (Encevis NeuroTrend) and had a predefined time limit of 5 min per EEG segment. All EEG segments were randomized and reviewed independently*.

* *p-Values of Kruskal-Wallis test after Bonferroni-Holmes correction for multiple testing; REV, reviewer*.

IRA of RPP showed substantial agreement for Reviewer #1 (Gwet's AC_1_ 0.72) and #3 (0.68) compared to the gold standard. Reviewer #2 (0.45) and #4 (0.54) showed moderate agreement (Table 2; *p* < 0.001 for all reviewers).

**Table 2:** Interrater agreement on the incidence of rhythmic and periodic EEG patterns in 76 continuous EEG segments of 20 critical care patients à 6 h.

	**Rhythmic and periodic EEG patterns**			
	**Gwet's AC_1_**	**95% C.I**.	*P*-Value^*^	**Agreement**
REV-1	0.72	0.59–0.86	<0.001	Substantial
REV-2	0.45	0.28–0.62	<0.001	Moderate
REV-3	0.68	0.54–0.82	<0.001	Substantial
REV-4	0.54	0.38–0.71	<0.001	Moderate

*Four board certified EEG reviewers used an automatic detection software (Encevis NeuroTrend) and had a predefined time limit of 5 min per EEG segment vs. gold standard (visual EEG analysis of three experienced EEG reviewers having unlimited time). All EEG segments were randomized and reviewed independently*.

* *p-Values of Chi-Square test after Bonferroni–Holmes correction for multiple testing; REV, reviewer*.

IRA of unequivocal ictal EEG patterns showed substantial agreement for all four reviewers compared to the gold standard. Sensitivity of individual reviewers ranged from 68.4 to 97.4% (mean 86.8%) and specificity from 68.4% to 92.1% (mean 82.2%) (Table 3; *p* < 0.001 for all reviewers).

**Table 3:** Sensitivity, specificity and interrater agreement on the incidence of unequivocal ictal EEG patterns in 76 continuous EEG segments of 20 critical care patients à 6 h.

	**Ictal EEG patterns**				
	**Gwet's AC_1_**	**95% C.I**.	**Sensitivity**	**Specificity**	*P*-Value^*^	**Agreement**
REV-1	0.71	0.55–0.87	92.1%	78.9%	<0.001	Substantial
REV-2	0.61	0.43–0.79	68.4%	92.1%	<0.001	Substantial
REV-3	0.79	0.65–0.93	89.5%	89.5%	<0.001	Substantial
REV-4	0.66	0.49–0.84	97.4%	68.4%	<0.001	Substantial

*Four board certified EEG reviewers used an automatic detection software (Encevis NeuroTrend) and had a predefined time limit of 5 min per EEG segment vs. gold standard (visual EEG analysis of three experienced EEG reviewers having unlimited time). All EEG segments were randomized and reviewed independently*.

* *p-Values of Chi-Square test after Bonferroni-Holmes correction for multiple testing; REV, reviewer*.

**Figure 3:**
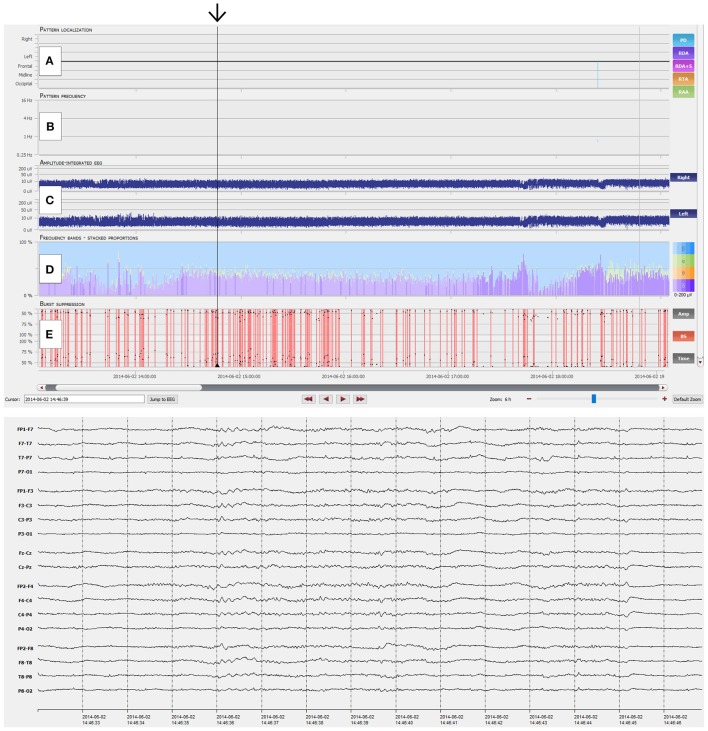
NeuroTrend example of a 49-year-old man with left temporal gliosis and sepsis. Six hours of continuous EEG (CEEG) are depicted with the Neurotrend GUI. Suppressed EEG due to sedoanalgesia can be clearly identified (black arrow). **(A)** No rhythmic or periodic EEG pattern was detected. **(B)** No pattern frequencies are displayed. **(C)** Amplitude integrated EEG shows a stable amplitude of 5–10 μV over both hemispheres. **(D)** Frequency bands show a low amplitude beta activity with underlying, low amplitude delta activity. **(E)** Burst suppression detection shows several periods with burst suppression. GUI, graphical user interface.

**Figure 4:**
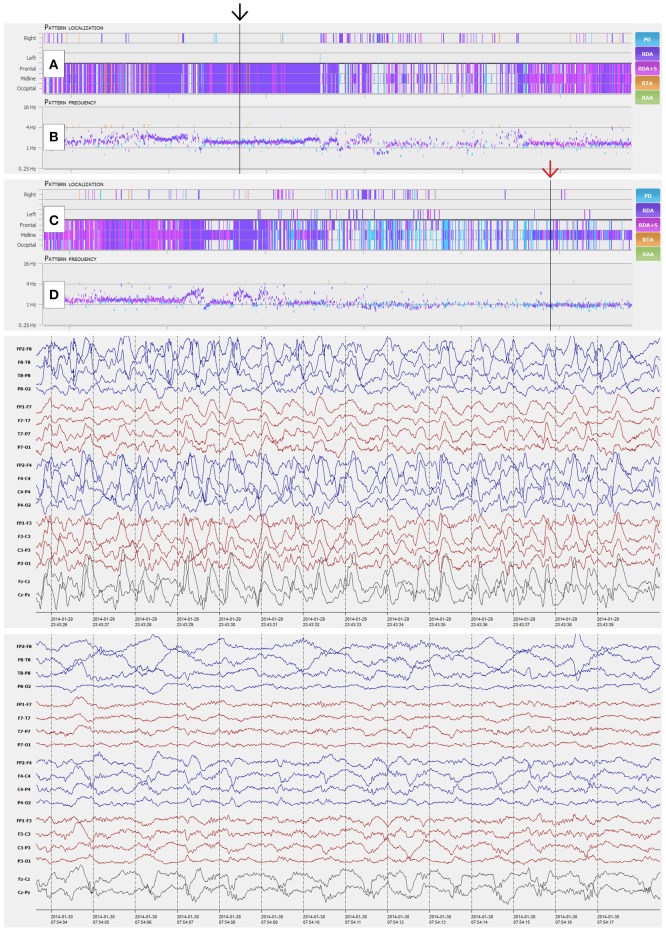
NeuroTrend examples of a 41-year-old woman with morphine abuse and sepsis. Six hours of continuous EEG (CEEG) are depicted with a compressed Neurotrend GUI in the top section (Amplitude integrated EEG, frequency bands, burst suppression detection, and heart rate frequency plot are hidden in this example). **(A**,**B)** display a stable detection of 1.5 c/s generalized rhythmic delta activity (GRDA, black arrow). The following 6 h of CEEG in the section below, show an overlap with a more periodic EEG pattern around 1 c/s after 3 h of recording (**C**,**D**, red arrow). GUI, graphical user interface.

IRA of burst-suppression showed substantial agreement for all four reviewers compared to the gold standard. Sensitivity of individual reviewers ranged from 93.3 to 100% (mean 96.7%) and specificity from 73.9 to 79.6% (mean 76.9%) (Table 4; *p* < 0.001 for all reviewers). Figures 3, 4 show examples of NeuroTrend detections.

**Table 4:** Sensitivity, specificity, and interrater agreement on the incidence of burst suppression in 76 continuous EEG segments of 20 critical care patients à 6 h.

	**Burst suppression**				
	**Gwet's AC_1_**	**95% C.I**.	**Sensitivity**	**Specificity**	*P*-Value^*^	**Agreement**
REV-1	0.69	0.52–0.85	93.3%	78.3%	<0.001	Substantial
REV-2	0.68	0.53–0.85	100%	73.9%	<0.001	Substantial
REV-3	0.71	0.56–0.87	96.7%	79.6%	<0.001	Substantial
REV-4	0.69	0.53–0.85	96.7%	76.1%	<0.001	Substantial

*Four board certified EEG reviewers used an automatic detection software (Encevis NeuroTrend) and had a predefined time limit of 5 min per EEG segment vs. gold standard (visual EEG analysis of three experienced EEG reviewers having unlimited time). All EEG segments were randomized and reviewed independently*.

* *p-Values of Chi-Square test after Bonferroni–Holmes correction for multiple testing; REV, reviewer*.

Unweighted MRA between reviewers showed moderate agreement regarding RPP (Gwet's AC_1_ 0.54; *p* = 0.07) and unequivocal ictal EEG patterns (0.57; *p* = 0.04). Almost perfect agreement was achieved for burst-suppression (0.86; *p* = 0.93; Table 5). It should be noted, that a high, non-significant difference in unweighted MRA analysis for binary items, emphasizes a very high agreement between all four reviewers.

**Table 5:** Unweighted multirater agreement (MRA) on the incidence of rhythmic and periodic EEG patterns, unequivocal ictal EEG patterns, and burst-suppression in 76 continuous EEG segments of 20 critical care patients à 6 h.

	**Unweighted MRA**			
	**Gwet's AC_1_**	**95% C.I**.	*P*-Value^*^	**Agreement**
Rhythmic and periodic EEG patterns	0.54	0.43–0.65	0.07	Moderate
Ictal EEG patterns	0.57	0.44–0.69	0.04	Moderate
Burst-suppression	0.86	0.77–0.94	0.93	Almost perfect

*Four board certified EEG reviewers used an automatic detection software (Encevis NeuroTrend) and had a predefined time limit of 5 min per EEG segment. All EEG segments were randomized and reviewed independently*.

* *p-Values of Chi-Square test after Bonferroni-Holmes correction for multiple testing*.

We included 45 CEEG datasets à 6 h of 15 critical care patients in our subanalysis of specific features of RPP (Main Terms and Modifiers according to the ACNS SCEET). Custom weighted MRA showed substantial agreement between reviewers for localization of RPP (Gwet's AC_2_ 0.65; *p* = 0.02), frequency of RPP (0.72; *p* < 0.001) and trend of RPP (0.74; p = 0.09). Moderate agreement was achieved for morphology of RPP (0.53; *p* < 0.001) and prevalence of RPP (0.56; *p* = 0.02; Table 6).

**Table 6:** Custom weighted multirater agreement (MRA) on the incidence of specific features of rhythmic and periodic EEG patterns in 45 continuous EEG segments of 15 critical care patients à 6 h.

	**Custom weighted MRA–subanalysis of specific EEG features**			
	**Gwet's AC_2_**	**95% C.I**.	*P*-Value^*^	**Agreement**
Localization (Main Term 1)	0.65	0.52–0.79	0.02	Substantial
Morphology (Main Term 2)	0.53	0.41–0.65	<0.001	Moderate
Prevalence	0.56	0.43–0.69	0.02	Moderate
Frequency	0.72	0.60–0.85	<0.001	Substantial
Trend	0.74	0.64–0.85	0.09	Substantial

*All four board certified EEG reviewers used an automatic detection software (Encevis NeuroTrend) and had a predefined time limit of 5 min per EEG segment. EEG segments were randomized and reviewed independently*.

* *p-Values of Chi-Square test after Bonferroni-Holmes correction for multiple testing*.

## Discussion

We conducted a multirater study to evaluate an automatic EEG pattern detection method (Encevis, NeuroTrend) for critical care CEEG in comparison to gold standard visual EEG analysis. Time limits were set to demonstrate the added value of NT.

### Review times

In general, very short review times (2.5–4 min per 6 h of CEEG; 9 to 15 min per 24 h of CEEG) were observed during our study, although there were statistical significant differences between individual reviewers. In comparison to a recent publication, which determined review times of various combinations of quantitative EEG (QEEG) and raw EEG analysis (QEEG only, 6 min; QEEG and raw EEG analysis, 14.5 min; raw EEG only, 19 min), our recorded review times were reasonable short (13). Another paper reported average review times of 8 min per 24 h of CEEG with compressed spectral array (CSA) guided review and 38 min with conventional visual EEG review. If seizures were present, prolonged review times were observed: 10 min for CSA and 44 min for conventional review (11). Other publications on automatic CEEG analysis did not report review times, although this a main point of interest (14, 16, 17).

### Rhythmic and periodic EEG patterns and ictal activity

Two reviewers showed substantial agreement for RPP in the IRA analysis. The other two reviewers had moderate agreement for RPP compared to the gold standard. Because RPP were a three point item (no pathologic EEG patterns according to ACNS SCEET Main Term #2 present; rhythmic or periodic EEG patterns according to ACNS SCEET Main Term #2 present; ictal EEG patterns according to current NCS criteria), unweighted agreement coefficient analysis was expected to be lower than in binary items. Also unweighted MRA showed only moderate agreement for RPP, meaning that the reviewers moderately matched in their annotations among each other. In the custom-weighted subanalysis of specific RPP (i.e., periodic discharges, rhythmic delta activity and spike-wave complexes) substantial MRA was found for localization, frequency and trend. Morphology and prevalence showed moderate agreement, reflecting the difficult assessment of these patterns. Due to our study design we could not report sensitivity and specificity of RPP detection. A previous publication reported high overall sensitivities of periodic epileptiform discharges (100%) and rhythmic delta activity (97.1%) with CSA guided review (11). Specificity and MRA was not assessed by the authors. To the best of our knowledge, other publications about automated critical care CEEG analysis did not assess RPP. We believe, that due to our strict time limits, the detailed assessment of difficult rhythmic and periodic EEG patterns was limited. However, we wanted to demonstrate that a straight-forward analysis of several hours of critical care CEEG is possible and feasible in a few minutes with our proposed automatic detection software.

We observed substantial IRA for unequivocal ictal EEG patterns with sensitivities ranging from 68 to 97% (mean 87%) and specificities from 68 to 92% (mean 82%), while MRA showed moderate agreement for ictal patterns. Our findings are in good agreement with previous studies, which used different QEEG techniques: overall sensitivities of seizure identification of 67–93%, specificities of 61–91% and false positive detection rates of 0.05–1 per hour were reported (11, 13–17). Low-amplitude, slow-frequency seizures which sometimes arise from RPP, seem to be harder to detect with automatic CEEG analysis, especially if RPP are continuously present (13). In our experience, automated, separate pattern detection results are very helpful in such demanding cases, but more review time may be needed, compared to clear cut high-frequency seizures.

We observed substantial IRA for burst suppression patterns with sensitivities ranging from 93 to 100% (mean 97%) and specificities from 74 to 80% (mean 77%). Kappa values of IRA were almost identical in a previous study conducted by our group, whereas sensitivity was lower and specificity slightly higher (29). Furthermore, MRA showed almost perfect agreement for burst suppression in the present study. This possibly reflects the good presentation of burst suppression patterns in the GUI of NT. In a recent survey, clinical neurophysiologists used automatic critical care CEEG analysis tools in 59% for burst suppression monitoring and in 29% for monitoring the depth of sedation (30). This findings emphasizes the need for a good performance of automatic burst suppression detection during critical care CEEG monitoring.

### Study design

We conducted an EEG-expert reader study to specifically evaluate the combined review approach of the NeuroTrend GUI with predefined time limits. NeuroTrend was developed and designed to use with two monitors with one screen for the automatic EEG pattern detection GUI and one screen for cross checking raw EEG (conventional review). This design intends to substantially reduce the workload of CEEG review by pre-filtering and categorizing relevant and important EEG information. Therefore, a study design was needed, which allowed independent EEG readers to annotate critical care continuous EEG with this specific review approach. To avoid possible reviewer bias, we did not conduct a second review and annotation round with conventional EEG analysis by the same four reviewers. This second review would not have been independent, because our review setup already included both automatic EEG pattern detection and conventional EEG review. Therefore, we compared individual annotations of the four included reviewers for each CEEG dataset with our defined gold standard (IRA) and among each other (MRA).

### Limitations

Our study has several limitations: First, training for our reviewers consisted of several steps but lasted just 2 h. Because all four reviewers were not familiar with the ACNS SCEET, which is currently not intended for regular clinical use, the learning curve may have been prolonged and might have affected annotations at the beginning of each reviewer. Longer training may provide higher agreement between reviewers and conventional EEG review (gold standard), especially for difficult, fluctuating rhythmic and periodic EEG patterns (17). Second, the predefined time limit for each CEEG dataset might have pushed the reviewers to hasty decisions. Based on CEEG review results, often critical decisions have to be made in intensive care patients and people with epilepsy on the ICU. Therefore it is not reasonable to limit CEEG review time in everyday clinical practice. However, if automatic CEEG pattern or seizure detection methods are scientifically tested without time limits, an added value is hard to prove. Third, compared to a previous publication on IRA of RPP using ACNS SCEET, our results showed lower agreement, sensitivity and specificity (25). The authors used snippets of EEGs (10 s to 1 min) to demonstrate the feasibility and reproducibility of SCCET Main terms and Modifiers. However, we focused on a straight-forward analysis of long term critical care EEG recordings with very short review times using an automatic EEG pattern detection method. Therefore, our results are reasonable from a clinical point of view.

## Conclusions

We provided evidence for the clinical feasibility of our proposed automatic EEG analysis software. It is a rapid and reasonable high sensitive review tool, but currently cannot replace raw EEG analysis and electrophysiological decision making in critical care patients due to the partly moderate specificity and interrater agreement. We observed very short review times, yet still reasonable high agreement for rhythmic and periodic EEG patterns, unequivocal ictal EEG patterns and burst suppression.

## Ethics statement

The study protocol was approved by the institutional ethics commission (Ethikkomission Medizinische Universität Wien, Ethikkommission der Stadt Wien). Informed consent was given by all reviewers, that volunteered for the study. Patients included in the study were mainly not able to give consent during continuous EEG recordings. Therefore, the ethics commission requested that all patients that were not able to give consent and their relatives receive a written patient information and/or were informed about the study and the possibility to withdraw their personal data in the future.

## Statistical testing

JK had full access to all the data in the study and takes responsibility for the integrity of the data and the accuracy of the data analysis.

## Author contributions

JK: study idea, study setup, study execution, statistical analysis, writing the manuscript, editing the manuscript; JH, FF, MH, TK, and CB: study idea, editing the manuscript; SP, VR-D, FR, and JF: study execution, editing the manuscript.

### Conflict of interest statement

FF, MH, and TK developed Encevis, NeuroTrend. JK, JH, and CB were involved in the development process of Encevis, NeuroTrend. The reviewer NM and handling Editor declared their shared affiliation. The remaining authors declare that the research was conducted in the absence of any commercial or financial relationships that could be construed as a potential conflict of interest.

## Abbreviations

ACNS: American Clinical Neurophysiology Society
CEEG: continuous electroencephalography
CCEEG: critical care continuous electroencephalography
CSA: compressed spectral array
EEG: electroencephalography
FN: false negative
FP: false positive
GUI: graphical user interface
ICU: intensive care unit
IRA: interrater agreement
MRA: multirater agreement
NCS: nonconvulsive seizures
NCSE: nonconvulsive status epilepticus
NT: NeuroTrend
PD: periodic discharges
QEEG: quantitative electroencephalography
RDA: rhythmic delta activity
RPP: rhythmic and periodic EEG patterns
SCCET: Standardized Critical Care EEG Terminology
SE: status epilepticus
SW: spike-and-wave complexes
TN: true negative
TP: true positive.
