# Supplementary material for: Automated Long-Term EEG Review: Fast and Precise Analysis in Critical Care Patients
Source: Front Neurol. 2018 Jun 19;9:454. doi: 10.3389/fneur.2018.00454 (PMC6020775; doi:10.3389/fneur.2018.00454)
Supplement: Supplementary file 1 [file Data_Sheet_1.DOCX]

**Reviewer:** **EEG-ID:**

| **Pattern #1**  **equals □ Status Epilepticus □ electrographic seizure □ no ictal activity** |
| --- |

| ***Localization*** | ***Morphology*** | ***Prevalence*** | ***Frequency*** | ***Trend*** |
| --- | --- | --- | --- | --- |
| G (generalized) | Electrographic seizure pattern (>4Hz) | >90% | > 3 Hz | evolution |
| L (lateralized) | SW  (Spike-and-Wave) | 50 – 89% | 1 – 3 Hz | fluctuation |
| Bi (Bilateral independent) | RDA  (rhythmic delta activity) | 10 – 49% | < 1 Hz | stationary |
|  | PD  (periodic discharges) | 1-9 % |  |  |

| **Pattern #2**  **equals □ Status Epilepticus □ electrographic seizure □ no ictal activity** |
| --- |

| ***Localization*** | ***Morphology*** | ***Prevalence*** | ***Frequency*** | ***Trend*** |
| --- | --- | --- | --- | --- |
| G (generalized) | Electrographic seizure pattern (>4Hz) | >90% | > 3 Hz | evolution |
| L (lateralized) | SW  (Spike-and-Wave) | 50 – 89% | 1 – 3 Hz | fluctuation |
| Bi (Bilateral independent) | RDA  (rhythmic delta activity) | 10 – 49% | < 1 Hz | stationary |
|  | PD  (periodic discharges) | 1-9 % |  |  |

| **Pattern #3**  **equals □ Status Epilepticus □ electrographic seizure □ no ictal activity** |
| --- |

| ***Localization*** | ***Morphology*** | ***Prevalence*** | ***Frequency*** | ***Trend*** |
| --- | --- | --- | --- | --- |
| G (generalized) | Electrographic seizure pattern (>4Hz) | >90% | > 3 Hz | evolution |
| L (lateralized) | SW  (Spike-and-Wave) | 50 – 89% | 1 – 3 Hz | fluctuation |
| Bi (Bilateral independent) | RDA  (rhythmic delta activity) | 10 – 49% | < 1 Hz | stationary |
|  | PD  (periodic discharges) | 1-9 % |  |  |

| **Burst-Suppression** **□ yes □ no**  **Slowing** **□ yes □ no**  *Localization* □ focal □ generalized  *Duration* □ intermittent □ continuous (>90%) |
| --- |
