# Supplementary material for: Automated Long-Term EEG Review: Fast and Precise Analysis in Critical Care Patients
Source: Front Neurol. 2018 Jun 19;9:454. doi: 10.3389/fneur.2018.00454 (PMC6020775; doi:10.3389/fneur.2018.00454)
Supplement: Supplementary file 2 [file Data_Sheet_2.DOCX]

We used Gwet’s multirater agreement coefficient AC_2_ for ordinal data to calculate custom weighted multirater agreement of specific features of rhythmic and periodic EEG patterns (according to ACNS standardized critical care EEG terminology) between all four reviewers in the following manner:

| 1. **Custom weights for Main Term #1 (Localization)** | | | | |
| --- | --- | --- | --- | --- |
|  | **no pattern present** | **generalized** | **lateralized** | **bilateral independent** |
| **no pattern present** | 1 | 0 | 0 | 0 |
| **generalized** | 0 | 1 | 0,6 | 0,8 |
| **lateralized** | 0 | 0,6 | 1 | 0,8 |
| **bilateral independent** | 0 | 0,8 | 0,8 | 1 |

| 1. **Custom weights for Main Term #2 (Morphology)** | | | | |
| --- | --- | --- | --- | --- |
|  | **no pattern present** | **spike-and-wave complexes** | **rhythmic delta activity** | **periodic discharges** |
| **no pattern present** | 1 | 0 | 0 | 0 |
| **spike-and-wave complexes** | 0 | 1 | 0,6 | 0,8 |
| **rhythmic delta activity** | 0 | 0,6 | 1 | 0,8 |
| **periodic discharges** | 0 | 0,8 | 0,8 | 1 |

| 1. **Custom weights for Modifier #1 (Prevalence)** | | | | | |
| --- | --- | --- | --- | --- | --- |
|  | **no pattern present** | **>90%** | **50-89%** | **10-49%** | **1-9%** |
| **no pattern present** | 1 | 0 | 0 | 0 | 0 |
| **>90%** | 0 | 1 | 0,8 | 0,6 | 0,4 |
| **50-89%** | 0 | 0,8 | 1 | 0,8 | 0,6 |
| **10-49%** | 0 | 0,6 | 0,8 | 1 | 0,8 |
| **1-9%** | 0 | 0,4 | 0,6 | 0,8 | 1 |

| 1. **Custom weights for Modifier #3 (Frequency)** | | | | |
| --- | --- | --- | --- | --- |
|  | **no pattern present** | **>3Hz** | **1-3Hz** | **<1Hz** |
| **no pattern present** | 1 | 0 | 0 | 0 |
| **>3Hz** | 0 | 1 | 0,8 | 0,6 |
| **1-3Hz** | 0 | 0,8 | 1 | 0,8 |
| **<1Hz** | 0 | 0,6 | 0,8 | 1 |

| 1. **Custom weights for Modifier #9 (Trend)** | | | | |
| --- | --- | --- | --- | --- |
|  | **no pattern present** | **evolution** | **fluctuation** | **stationary** |
| **no pattern present** | 1 | 0 | 0 | 0 |
| **evolution** | 0 | 1 | 0,8 | 0,6 |
| **fluctuation** | 0 | 0,8 | 1 | 0,6 |
| **stationary** | 0 | 0,6 | 0,6 | 1 |
